# USE OF EXPOSURE CROSSOVER DESIGN TO CONTROL FOR UNMEASURED BASELINE CONFOUNDING IN OBSERVATIONAL STUDIES

**DOI:** 10.1093/geroni/igz038.1785

**Published:** 2019-11-08

**Authors:** Ling Han, Robert D Kerns, Melissa Skanderson, Joseph L Goulet, Stephen Luther, Cynthia Brandt

**Affiliations:** 1 Yale School of Medicine, New Haven, Connecticut, United States; 2 Yale University, New Haven, Connecticut, United States; 3 VA Connecticut, West Haven, Connecticut, United States; 4 VA Connecticut Healthcare System, West Haven, Connecticut, United States; 5 James A. Haley Veterans Hospital, Tampa, Florida, United States

## Abstract

Observational comparative effectiveness studies face the challenge of selection bias. Due to lack of randomization, an alleged treatment effect may reflect inherent differences in baseline characteristics between comparison groups, rather than the outcome of treatment. Propensity score methods were devised to “resample” a most comparable comparison group, under a strong yet untestable assumption of no unmeasured confounding. We present an “exposure crossover” study evaluating complementary and integrative health approaches (CIH) among 6,379 US veterans who received acupuncture, massage or chiropractic therapies between 10/1/2011-9/30/2013. Their average pain intensity ratings (PIRs) during the 12-months after CIH initiation (effect period, EP) were compared with the 12-months before (baseline period, BP). Through this built-in self-matching, veterans’ characteristics and other stable baseline confounding, measured and unmeasured, were presumably eliminated. After accounting for time-varying opioid use and within-subject correlations using a generalized estimating equation, we found that in comparison to the BP, the adjusted mean PIR during the EP was -0.40 (95% Confidence Interval (CI): -0.51, -0.29) points lower; while the adjusted rate ratio of moderate to severe pain (PIRs ≥ 4) was 34% lower [0.66 (95% CI: 0.62, 0.70)]. The effect sizes were greater among veterans older than 65 years, yet diminished to null after 6-9 months. Assuming a 3-month induction period, using alternative random-intercept model, and examining post-CIH opioid use as an alternative outcome, derived similar results. These observations echo some randomized trials suggesting a modest, short-term CIH benefit, and highlight the merits and usefulness of exposure-crossover design to observational studies of medical interventions.

